# Severe cellular stress activates apoptosis independently of p53 in osteosarcoma

**DOI:** 10.1038/s41420-021-00658-y

**Published:** 2021-10-04

**Authors:** Cheng-Jung Ho, Huey-Jiun Ko, Tzu-Shao Liao, Xiang-Ren Zheng, Po-Hsu Chou, Li-Ting Wang, Ru-Wei Lin, Chung-Hwan Chen, Chihuei Wang

**Affiliations:** 1 Department of Orthopedics, Kaohsiung Medical University Hospital, Kaohsiung, 80708 Taiwan; 2 Graduate Institute of Clinical Medicine, Kaohsiung Medical University, Kaohsiung, 80708 Taiwan; 3 Department of Biochemistry & Graduate Institute of Medicine, Kaohsiung Medical University, Kaohsiung, 80708 Taiwan; 4 Department of Biotechnology, Kaohsiung Medical University, Kaohsiung, 80708 Taiwan; 5 Department of Plant Industry, National Pingtung University of Science and Technology, Pingtung, 91201 Taiwan; 6 Department of Medical Research, Kaohsiung Medical University Hospital, Kaohsiung, 80708 Taiwan

**Keywords:** Apoptosis, Chemotherapy

## Abstract

Apoptosis induced by doxorubicin, bortezomib, or paclitaxel, targeting DNA, 26S proteasome, and microtubules respectively, was assessed in two osteosarcoma cells, p53 wild-type U2OS and p53-null MG63 cells. Doxorubicin-induced apoptosis only occurred in U2OS, not in MG63. In contrast, bortezomib and paclitaxel could drive U2OS or MG63 toward apoptosis effectively, suggesting that apoptosis induced by bortezomib or paclitaxel is p53-independent. The expressions of Bcl2 family members such as Bcl2, Bcl-xl, and Puma could be seen in U2OS and MG63 cells with or without doxorubicin, bortezomib, or paclitaxel treatment. In contrast, another member, Bim, only could be observed in U2OS, not in MG63, under the same conditions. Bim knockdown did not affect the doxorubicin-induced apoptosis in U2OS, suggested that a BH3-only protein other than Bim might participate in apoptosis induced by doxorubicin. Using a BH3-mimetic, ABT-263, to inhibit Bcl2 or Bcl-xl produced a limited apoptotic response in U2OS and MG63 cells, suggesting that this BH3-mimetic cannot activate the Bax/Bak pathway efficiently. Significantly, ABT-263 enhanced doxorubicin- and bortezomib-induced apoptosis synergistically in U2OS and MG63 cells. These results implied that the severe cellular stress caused by doxorubicin or bortezomib might be mediated through a dual process to control apoptosis. Respectively, doxorubicin or bortezomib activates a BH3-only protein in one way and corresponding unknown factors in another way to affect mitochondrial outer membrane permeability, resulting in apoptosis. The combination of doxorubicin with ABT-263 could produce synergistic apoptosis in MG63 cells, which lack p53, suggesting that p53 has no role in doxorubicin-induced apoptosis in osteosarcoma. In addition, ABT-263 enhanced paclitaxel to induce moderate levels of apoptosis.

## Introduction

Osteosarcoma is the most common malignant primary bone tumor in children and adolescents. Prior to the 1950s, the treatment of osteosarcoma depended mainly on surgical excision of the tumor, and the 5-year survival rates were less than 20%. In the 1970s, with the combination of neoadjuvant chemotherapy, tumor wide excision, and adjuvant chemotherapy, the 5-year survival rates improved to 60–70%. However, in the subsequent 40 years, the survival rates for osteosarcoma plateaued [1, 2], despite improvements in diagnostic imaging, surgical techniques, reconstruction instruments, and the mapping of the whole human genome. The development of distant metastasis, especially pulmonary metastasis, is the main cause of osteosarcoma treatment failure. It is assumed that around 20% of osteosarcoma patients have detectable metastasis at diagnosis [3]. The overall survival of osteosarcoma patients with lung metastasis is extremely low, with only 30% 5-year-survival.

The expressions of mutated p53, c-myc, and Bcl2 are correlated to poor prognosis in osteosarcoma patients [4]. Most osteosarcomas bear nonfunctional *TP53*, *RB* mutation, or increasing copy numbers of *MDM2/4* [5, 6]. The inactivation of *Trp53* and *RB* develops metastatic osteosarcoma in mouse osteoblast lineage [7]. Both *TP53* and *RB* are tumor suppressor genes. RB functions as a transcriptional co-factor that antagonizes or potentiates the activities of numerous transcription factors, which mainly control cell cycle progression [8]. Different from RB, p53 works as a transcription factor involved in the expression of about 500 target genes responsible for cell cycle arrest, cell senescence, DNA repair, metabolic adaptation, and cell death [9]. Thus, p53 is viewed as a master guardian for maintaining genome integrity and for driving severely damaged cells toward death, accounting for its tumor suppression role [10]. It also participates in cancer therapy [11].

The drugs used for cancer chemotherapy mainly act by the induction of apoptotic pathways. The driving force of apoptosis is the cellular stress generated by therapeutic drugs that mostly target DNA or microtubules to cause either severe DNA damage (SDD) or spindle mis-assembly (SM) stress. The sensors of cellular stresses induce the Bcl2 family to generate mitochondrial outer membrane permeability (MOMP), resulting in apoptosis [12]. The most well-known sensor is p53 [9]. p53 can activate the expression of BH3-only proteins such as Puma, Noxa, or Bim to counteract the repression effect of antiapoptotic proteins on pro-apoptotic Bax/Bak proteins for the formation of MOMP [13–15]. Cancer cells with wild-type *TP53* demonstrate higher sensitivity to chemotherapy agents than cancer cells with mutated or no *TP53* [16].

The agents used for osteosarcoma mostly target DNA, including doxorubicin (DOX), cisplatin, epirubicin, ifosfamide, cyclophosphamide, etoposide, gemcitabine, topotecan, and an antimetabolite, methotrexate [17–19]. They can be used as single agents but are mostly employed in combination [18, 19]. In addition, microtubule-targeting agents including Vinca alkaloids and taxanes have been used for second-line chemotherapy in high-grade osteosarcoma [20]. Over the past two decades, no new agents have been added to treatment protocols for osteosarcoma.

Recently, many new chemotherapy agents, which interact with targets other than DNA and microtubules, have been developed to treat cancers. Of these, two groups of compounds corresponding to BH3-mimetics and ubiquitin-proteasome pathway (UPP) inhibitors demonstrate great potency for cancer therapy. The rational design of BH3-mimetics is to bypass the resistant phenotype of cancer cells caused by the silencing of upstream regulators of BH3-only proteins such as p53 [21]. Representative BH3-mimetics include ABT-737 and its clinical analog ABT-263 (brand name Navitoclax), which mimic the BH3 domain of Bim and can interact with Bcl2, Bcl-xl, or Bcl-W antiapoptotic proteins [22]. ABT-737 can sensitize cisplatin-induced apoptosis in the U2OS osteosarcoma cell line [23]. Regarding UPP inhibitors, the most representative agent is bortezomib (BTZ). This compound has been proven to treat multiple myeloma and mantle cell lymphoma [24, 25]. BTZ interacts with the 20S catalytic core of the 26S proteasome to block UPP, impeding protein degradation to generate protein turnover dysfunction (PTD) stress [26]. The inactivation of NF-κB, the stabilization of p53, proapoptotic proteins or BH3 only proteins, the depletion of ubiquitin, the increase of ER stress or JNK pathway action due to the disruption of protein degradation are all possible reasons for BTZ-induced apoptosis [27, 28]. BTZ has also great efficacy for triggering apoptosis in osteosarcoma cells [29].

In this study, we analyzed the apoptotic response of the p53 wild-type U2OS and p53-null MG63 osteosarcoma cell lines to SDD, PTD, and SM. We demonstrated that PTD or SM, induced by BTZ or paclitaxel (PTX), respectively, drive significant apoptosis in both U2OS and MG63 cells. In contrast, the effective apoptosis for DOX-induced SDD only appeared in U2OS, not in MG63. In addition, we asked if ABT-263 could trigger apoptosis in U2OS and MG63 cells without cellular stress. The result showed that ABT-263 induces an only limited apoptotic response in U2OS and MG63 in the absence of cellular stress. We then combined ABT-263 with BTZ, DOX, or PTX to treat U2OS and MG63, and we revealed that ABT-263 synergizes DOX- and BTZ-induced apoptosis. The enhancing effect of ABT-263 on PTX apoptosis is much less than its effect with DOX or BTZ. This synergistic effect indicated that ABT-263 might couple with the SDD or PTD driving forces to activate the apoptosis pathway. However, ABT-263 and SM might work independently to produce complicated programmed cell death. DOX-, BTZ-, and PTX-induced apoptosis seemed not to involve p53.

## Results

### DOX-, BTZ-, or PTX-induced apoptosis in U2OS and MG63 cells

**Fig. 1:**
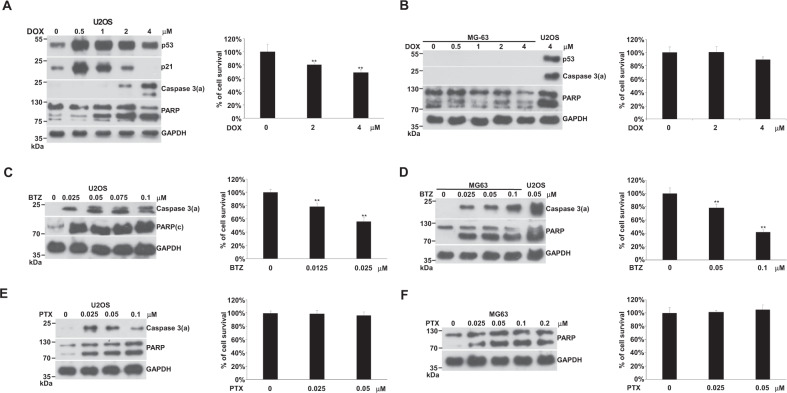
Effects of DOX, BTZ, and PTX on apoptosis in U2OS and MG63 cells. Cells were incubated with various concentrations of DOX, BTZ, or PTX in 10-cm plates or 96-well plates for 24 h and harvested for western blotting or cell survival assay, respectively. The immunoblot images of p53, p21, caspase 3(a), or PARP were shown. GAPDH served as a loading control. The percentages of cell survival normalized to the cells without compound treatment were shown beside the right side of the immunoblot image (**P < 0.01). The immune blots were cropped from different parts of the same gels and visualized by ECL under various exposure conditions dependent on the activity of antibodies. Representative blots from triplicate experiments were shown. **A** U2OS was treated with DOX. **B** MG63 treated with DOX. **C** U2OS treated with BTZ. **D** MG63 treated with BTZ. **E** U2OS treated with PTX. **F** MG63 treated with PTX.

We compared the apoptotic response of U2OS and MG63 cells to DOX. DOX-induced apoptosis at 1 μM to achieve significant PARP degradation in U2OS (Fig. 1A). When the DOX concentration was increased to 2 μM, the activation form of caspase 3, caspase 3(a), became visible and the percentage of cell survival decreased (Fig. 1A). Significant apoptosis and cell death appeared at 4 μM (Fig. 1A). Two events, p21 expression, and activation of caspase 3 can be separated correspondingly to low and high concentrations of DOX (Fig. 1A). The level of p53 highly matched the expression of p21, but it was inversely related to the extent of apoptosis. In MG63, which lacks p53, caspase 3(a) and significant cell death could not be detected even with 4 μM DOX (Fig. 1B).

In contrast, BTZ triggered apoptosis in both U2OS and MG63 cells efficiently, based on the level of caspase 3(a) (Fig. 1C, D). In U2OS, apoptosis could be activated by 0.025 μM of BTZ and reached to a maximum at 0.05 μM. The intensity of apoptosis matched the extent of cell death in both cells (Fig. 1C, D). MG63 was less sensitive than U2OS in response to BTZ though its apoptotic response kept rising along with increasing concentrations of BTZ (Fig. 1D). Both cells had a very good responses to BTZ treatment, possibly due to their high proliferation rate requiring hasty protein turnover. Once PTD occurs, it might become enough cellular stress to initiate apoptosis.

The concentration of PTX required to activate apoptosis was low, about 0.025 μM in both U2OS and MG63 cells (Fig. 1E, F). U2OS was more sensitive than MG63 in response to PTZ. Saturation was reached at about 0.025 or 0.05 μM for U2OS or MG63, respectively (Fig. 1E, F). No cell death was detected in either cell line (Fig. 1E, F). The disruption of microtubule dynamics might be the reason for PTX apoptosis [30]. A recent report found that the mitotic checkpoint can activate NEK2A and separase to cleave the N-terminal of MCL1 in response to SM, resulting in the C-terminal half of MCL1 forming Bax/Bak-like pores [31]. Just like Bax/Bak pores, pores formed by the C-terminal half of MCL1 might also generate MOMP and subsequently apoptosis.

From the above results, we could not conclude whether p53 is involved in DOX-induced apoptosis or not. However, both BTZ- and PTX-induced apoptosis obviously occurred through a p53-independent pathway.

### The expression of Bcl2, Bcl-xl, Bim, and Puma in U2OS and MG63 cells

**Fig. 2:**
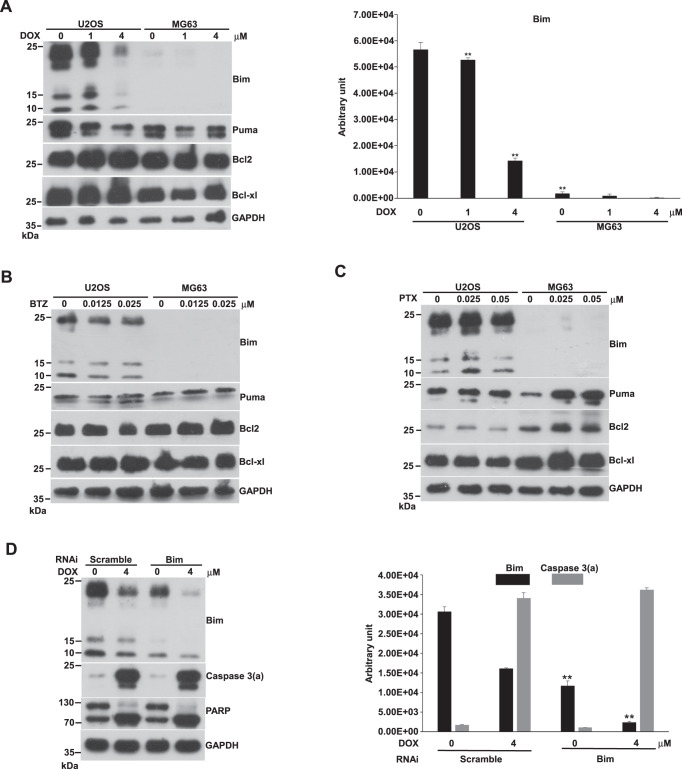
Expression of Bcl2, Bcl-xl, Bim, and Puma in U2OS and MG63 cells with/without DOX, BTZ, and PTX treatment and the knockdown of Bim in U2OS cells. Cells were incubated with various concentrations of DOX, BTZ, or PTX on 10-cm plates for 24 h and harvested for western blotting. The immunoblot images of Bcl2, Bcl-xl, Bim, and Puma were shown. GAPDH served as a loading control. The immunoblot images of Bim normalized to GAPDH were quantitated as shown beside the right side of the immunoblot image in DOX-treated U2OS and MG cells (**P < 0.01). For Bim knockdown, U2OS cells were transfected with the Bim specific or scramble RNAi and then were treated with 0 and 4 μM of DOX for 24 h. Cells were harvested for western blotting. The immunoblot images of Bim and caspase 3(a) were shown. GAPDH served as a loading control. The immunoblot images of Bim and caspase 3(a) normalized to GAPDH were quantitated as shown beside the right side of the immunoblot image (**P < 0.01). The immune blots were cropped from different parts of the same gels and visualized by ECL with various exposure conditions dependent on antibody activity. Representative blots from triplicate experiments were shown. **A** U2OS and MG63 cells were treated by DOX. **B** U2OS and MG63 cells treated by BTZ. **C** U2OS and MG63 cells treated by PTX. **D** The Bim knockdown in U2OS.

The expression of Bcl2, Bcl-xl, and Puma can be detected in U2OS and MG63 cells with/without DOX, BTZ, or PTX treatment (Fig. 2A–C). The amounts of them did not change by increasing concentrations of compounds. A previous study has claimed that Puma can be activated transcriptionally by p53. It might be not the case in osteosarcoma.

Bim could only be detected in U2OS, not in MG63, and its level remarkably decreased with the increasing DOX concentration (Fig. 2A). Since Bim is the BH3-only protein responsible for DOX-induced apoptosis in prostate cancer [32], it might play the same role in osteosarcoma. Knockdown of Bim did not affect the DOX-induced apoptosis in U2OS cells (Fig. 2D). Different from prostate cancer, a BH3-only protein other than Bim might be responsible for the DOX-induced apoptosis in U2OS.

### ABT-263-induced apoptosis in U2OS and MG63 cells

**Fig. 3:**
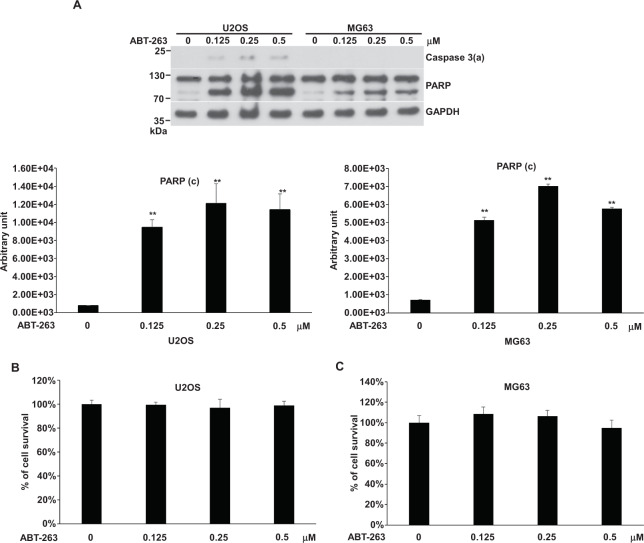
Effects of ABT-263 on apoptosis in U2OS and MG63 cells. Cells were incubated with various concentrations of ABT-263 in 10-cm plates or 96-well plates for 24 h and harvested for western blotting or cell survival assay, respectively. The immunoblot images of caspase 3(a) and PARP were shown. GAPDH served as a loading control. Immunoblot images of PARP (c), the cleavage PARP, normalized to GAPDH were quantitated as shown below immunoblot image (**P < 0.01). The percentages of cell survival normalized to the cells without compound treatment were shown. The immune blots were cropped from different parts of the same gels and visualized by ECL with various exposure conditions dependent on the activity of antibodies. Representative blots from triplicate experiments were shown. **A** Immunoblot image of U2OS and MG63. **B** U2OS cell survival. **C** MG63 cell survival.

With the high expression of Bcl2 and Bcl-xl in U2OS and MG63 cells, we asked if ABT-263, which interacts with Bc2 or Bcl-xl with high affinity, can induce apoptosis in the absence of cellular stress. Interestingly, ABT-263 could only induce a low degree of apoptosis in MG63 and U2OS cells (Fig. 3A). The capacity of ABT-263 to generate apoptosis did not increase after its concentration reached 0.25 μM (Fig. 3A). No cell death was shown in both cells (Fig. 3B, C). Based on the rational design of ABT-263, it should trigger apoptosis proficiently in the absence of cellular stress. Our results, however, seemed not to support this point. Thus, we further tested whether cellular stress such as SDD, PTD, or SD can enhance ABT-263-induced apoptosis in MG63 and U2OS.

### The combination of DOX with ABT-263 drove apoptosis synergistically in U2OS and MG63 cells

**Fig. 4:**
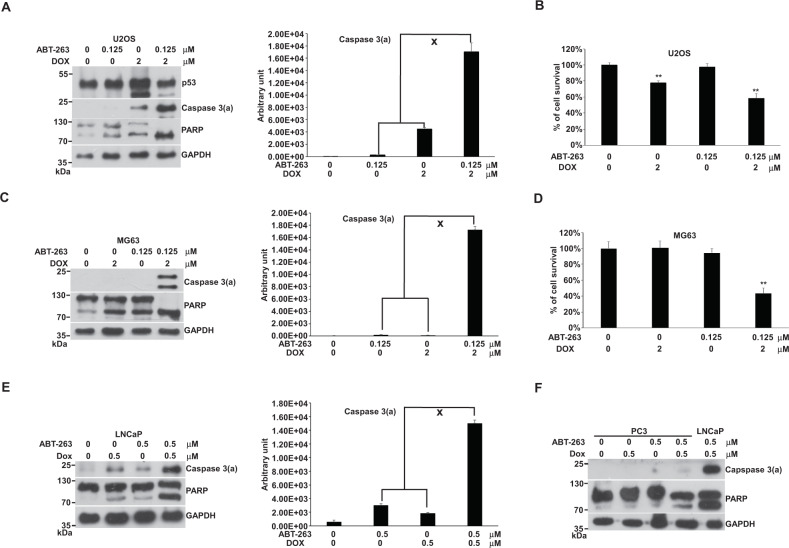
Effects of the combination of DOX with ABT-263 on apoptosis in U2OS, MG63, LNCaP, and PC3 cells. Cells were incubated with ABT-263, DOX, or a combination of both in 10-cm plates or 96-well plates for 24 h and harvested for western blotting or cell survival assay, respectively. The immunoblot images of p53 of U2OS and the immunoblot images of caspase 3(a) and PARP of U2OS and MG63 were shown. GAPDH served as a loading control. The immunoblot images of caspase 3(a) normalized to GAPDH were quantitated as shown beside the right side of the immunoblot image. The “x” indicated a synergistic outcome. The percentages of cell survival normalized to the cells without compound treatment were shown (**P < 0.01). The immune blots were cropped from different parts of the same gels and visualized by ECL with various exposure conditions dependent on the activity of antibodies. Representative blots from triplicate experiments were shown. **A** Immunoblot image of U2OS. **B** U2OS cell survival. **C** Immunoblot image of MG63. **D** MG63 cell survival. **E** Immunoblot image of LNCaP. **F** Immunoblot image of PC3.

We first combined DOX with ABT-363 to treat U2OS and MG63 cells. The results demonstrated a synergistic effect on apoptosis and cell death in U2OS and MG63 (Fig. 4A–D). ABT-263 seemingly interacted with Bcl2 or Bcl-xl to cooperate with the SDD-induced driving force, resulting in synergistic apoptosis. Since MG63 does not have functional *p53*, we thought that p53 is not involved in this SDD-driven force. Both U2OS and MG63 have the machinery responsible for this SDD-driven force.

Previously, we assessed the effect of ABT-263 on LNCaP and PC3 prostate cancer cells, which are p53-wild type and p53-null, respectively. We showed that the combination of DOX with ABT-263 displays synergistic apoptosis in LNCaP [33]. ABT-263 alone or combined with DOX can induce apoptosis at a relatively low level in PC3 in comparison with LNCaP [32]. Here, we re-evaluated the effect of ABT-263 on apoptosis in LNCaP and PC3 in single-agent and combination format. The results demonstrated that the synergistic apoptosis induced by the combination of DOX with ABT-263 only appears in LNCaP, not in PC3 (Fig. 4E, F). Unlike MG3, PC3 cells might not have the machinery for SDD-driven effects. Same as U2OS and MG63, LNCaP cells might bear this machinery.

### The combination of BTZ with ABT-263 drove apoptosis synergistically in MG63 and U2OS cells

**Fig. 5:**
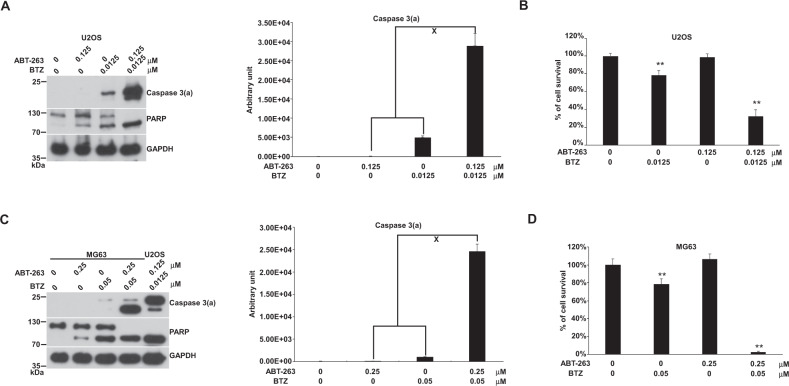
Effects of the combination of BTZ with ABT-263 on apoptosis in U2OS and MG63 cells. Cells were incubated with ABT-263, BTZ, or a combination of both in 10-cm plates or 96-well plates for 24 h and harvested for western blotting or cell survival assay, respectively. The immunoblot images of caspase 3(a) and PARP and cell death results were shown. GAPDH served as a loading control. The immunoblot images of caspase 3(a) normalized to GAPDH in cells were quantitated as shown. The “x” indicated a synergistic outcome. The percentages of cell survival normalized to the cells without compound treatment were shown (**P < 0.01). The immune blots were cropped from different parts of the same gels and visualized by ECL with various exposure conditions dependent on the activity of antibodies. Representative blots from triplicate experiments were shown. **A** Immunoblot image of U2OS. **B** U2OS cell survival. **C** Immunoblot image of MG63. **D** MG63 cell survival.

BTZ causes PTD and was a very potent compound for inducing apoptosis in both U2OS and MG63 cells. Interestingly, the combination of BTZ with ABT-263 showed a high synergistic effect on apoptosis and cell death in U2OS and MG63 cells (Fig. 5A–D). These results indicated that a BH3-only protein might participate in PTD-induced apoptosis. ABT-263 bound to Bcl2 or Bcl-xl to couple with a PTD-driving force to maximize apoptosis.

### ABT-263 enhanced PTX-induced apoptosis in U2OS and MG63 cells

**Fig. 6:**
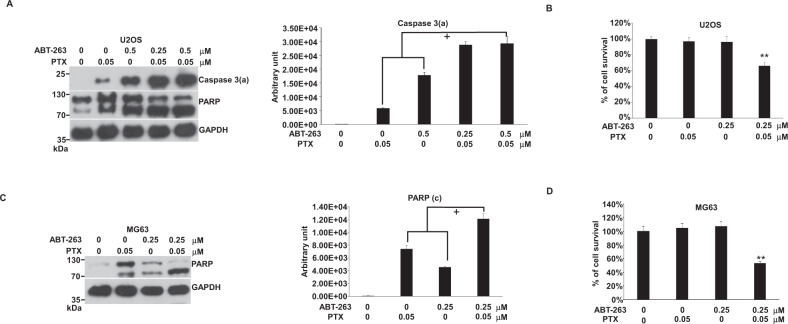
Effects of the combination of PTX with ABT-263 on apoptosis in U2OS and MG63 cells. Cells were incubated with ABT-263, PTX, or a combination of both in 10-cm plates or 96-well plates for 24 h and harvested for western blotting or cell death assay, respectively. The immunoblot images of caspase 3(a) and PARP and cell death results were shown. GAPDH served as a loading control. The immunoblot images of caspase 3(a) or PARP normalized to GAPDH in cells were quantitated as shown. The “+” indicated an additive outcome. The percentages of cell survival normalized to the cells without compound treatment were shown (**P < 0.01). Immune blots were cropped from different parts of the same gels and visualized by ECL with various exposure conditions dependent on the activity of antibodies. Representative blots from triplicate experiments were shown. **A** Immunoblot image of U2OS. **B** U2OS cell survival. **C** Immunoblot image of MG63. **D** MG63 cell survival.

The SM caused by PTX might not occur through the Bax/Bak pathway to activate apoptosis [31]. ABT-263 and PTX likely worked independently to drive apoptosis in combination. Based on the results of immunoblotting, the enhancing effect of ABT-263 for PTX appeared to be additive in U2OS or MG63 cells (Fig. 6A, C). Moreover, the combination of PTX with ABT-263 might shorten the timing for cells entering into the late apoptosis phase, based on the cell survival percentage (Fig. 6B, D).

### The translocation of p53 into the nucleus for responding to DNA damage in U2OS cells

**Fig. 7:**
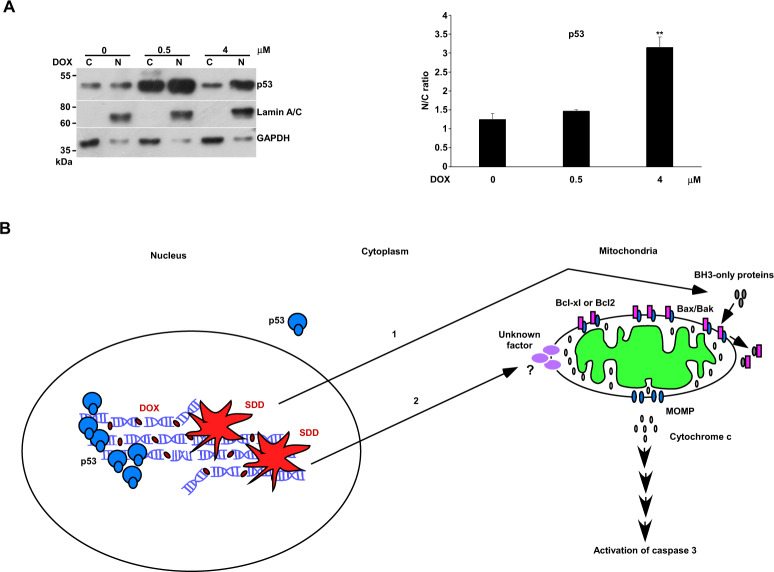
p53 localization and the diagram of the dual control model. U2OS cells were incubated with various concentrations of DOX in 10-cm plates and harvested for nuclei and cytosol fractionation. The nuclei and cytosol fraction were analyzed by western blotting. The immunoblot image of p53 was shown. Lamin A/C and GAPDH were served as the specific markers of nuclei and cytosol, respectively. The ratio of p53 in nuclei to p53 in the cytosol was quantitated as shown (**P < 0.01). N: nucleus. C: cytoplasm. Immune blots were cropped from different parts of the same gels and visualized by ECL with various exposure conditions dependent on the activity of antibodies. Representative blots from triplicate experiments were shown. **A** p53 localization. **B** SDD effect on apoptosis is mediated through a dual control pathway.

Our results indicated that p53 might not participate in SDD-induced apoptosis. Indeed, we saw that the level of p53 decreased along with apoptosis occurring (Fig. 1A). We compared the localization of p53 in no apoptosis with that in apoptosis. The result demonstrated that the ratio of p53 in nuclei to p53 in the cytosol was higher in apoptosis than that in no apoptosis, even a much higher level of p53 in no apoptosis than in apoptosis (Fig. 7A). This result indicated that the translocation of p53 into the nucleus kept going as DNA damage proceeded, and SDD just activated apoptosis in its own way, obviously no linking to p53 (Fig. 7B).

## Discussion

This study suggested that inhibitors of UPP and BH3-mimetics such as BTZ and ABT-263 have great potential as chemotherapy for osteosarcoma. The combination of ABT-263 with other agents including DOX, BTZ, and PTX greatly promoted cell killing in osteosarcoma. More importantly, we provided strong evidence that p53 might play no role in DOX-, BTZ-, and PTX-induced apoptosis.

As a transcription factor, p53 can activate the expression of Puma, Noxa, and Bim to initiate apoptosis. It might translocate into mitochondria to propel apoptosis through a transcription-independent pathway [34, 35]. DNA targeting agent-induced cell death is believed to mostly occur through p53-mediated apoptosis. However, the functions of p53 in taxane- or UPP inhibitor-induced apoptosis remain controversial.

Treating LNCaP and PC3 prostate cancer cells with DOX, we have shown that Bim counteracts Bcl-xl to induce apoptosis in a p53-independent manner [32]. Bim and Puma constantly exist in LNCaP and PC3 with/without DOX, inconsistent with previous studies. The threshold of apoptosis is determined by SDD, not the amount of p53 [33], although one study claims that the apoptotic threshold is determined by expression levels of p53 and its targets [36]. A dominant-negative p53 reduces the expression of p21 and has no effect on DOX-induced apoptosis, suggested that the transcription activity of p53 is not related to apoptosis caused by SDD [37].

As in prostate cancer, we demonstrated that the transcriptional activity of p53 did not contribute to DOX-induced apoptosis in glioblastoma [38]. Moreover, our results showed that p53 translocation into the nucleus corresponds with DNA damage other than apoptosis, consistent with the current study. Using a natural product, sanguinarine, which causes DNA single and double-strand breaks, one study found that sanguinarine-induced apoptosis is p53-independent in human colon cancer cell lines [39]. Another publication has shown that embryonic stem cells do not activate p53-dependent stress responses and undergo p53-independent apoptosis in response to DNA damage [40].

In the current study, we showed that ABT-263 has a limited effect on apoptosis in U2OS and MG63 cells. This result suggested that ABT-263 cannot activate the Bax/Bak-dependent pathway efficiently and thus implied the dual control of this pathway. This means that cellular stress such as SDD induced by DOX will activate a BH3-only protein in one way alongside an unknown factor in another way to affect MOMP and subsequent apoptosis (Fig. 7B). The translocation of p53 into the nucleus was related to DNA damage rather than apoptosis. PTD induced by BTZ will work just as DOX did. it might also excite a BH3-only protein to couple with another driving force to produce apoptosis. Thus, the combination of DOX or BTZ with ABT-263 generated synergistic apoptosis in U2OS and MG63. ABT-263 here worked as an inhibitor of Bcl2 or Bcl-xl, suggested that DOX could not effectively produce apoptosis in MG63 due to having no specific BH3-only protein to counteract with Bcl2 or Bcl-xl. With the addition of ABT-263, DOX could produce profound apoptosis in p53-null MG63, indicating that p53 has no role in SDD-induced apoptosis.

The magnitude of apoptosis induced by the combination of PTX with ABT-263 was much less than that from combining ABT-263 with DOX or BTZ. The reason is probably that PTX action in apoptosis does not happen through a Bax/Bak-dependent pathway. Thus, there was no coupling action to generate a synergistic effect. However, PTX action seemed to take place in mitochondria to create MOMP. Thus, we still saw a great enhancing effect of ABT-263 on PTX-induced apoptosis and cell death.

All in all, our studies excluded the role of p53 in SDD, PTD, and SM-induced apoptosis in osteosarcoma. The main function of p53 is to protect the integrity of the genome from DNA damage. Thus, the genes involved in apoptosis in response to SDD, PTD, and SM might be lost if p53 is mutated or absent. This probably is a reason why cancer cells with missing or mutated p53 always display chemotherapy-resistant phenotypes.

## Materials and methods

### Compounds

DOX (Merck Millipore, Burlington, MA, USA), BTZ (Selleck, Houston, TX, USA), PTX (Merck Millipore), and ABT-263 (Selleck) were purchased as indicated. Compounds were dissolved in DMSO to prepare the stock solution and the stock was stored at −20 °C before use.

### Cell culture and compound treatment

Two osteosarcoma cell lines, U2OS and MG63, and two prostate cancer cell lines, LNCaP and PC3, were obtained from the Bioresource Collection and Research Center in Taiwan. Cells were cultured in an incubator at 37 °C under 5% CO_2_. The medium for U2OS and MG63 was McCoy’s 5A (HyClone, Logan, UT, USA) and EMEM (HyClone) with 10% fetal bovine serum, respectively. The medium for LNCaP and PC3 was RPMI-1640 (HyClone) with 10% fetal bovine serum. About 1–2 × 10^6^ cells were seeded on Petri dishes (10 cm). When cell growth reached 70–80% confluence, the old medium was replaced by a fresh medium. The cells were treated with various concentrations of compounds singly or in combinations for 24 h. After treatment, the cells were harvested, washed with PBS, and spun down.

### Bim knockdown by RNAi

U2OS cells were grown up to 60% confluence. Bim siRNA (Santa Cruz Biotechnology, sc-29802, Dallas, TX, USA) or control siRNA (Santa Cruz Biotechnology, sc-37007) was pre-incubated in 500 μl culture medium (no serum) before transfection, and then 40 μl INTERFERin transfection reagent (Polyplus, 409-10, Illkirch, France) was added to this culture medium, mixed by vortex and incubated for 10 min at room temperature to allow the formation of transfection complexes. The siRNA complex was added dropwise to U2OS cells on a 10 cm dish culture. After 12 h, the old medium was replaced by a fresh medium containing 0 or 4 μM of DOX. The cells were harvested for immunoblotting after 24 h.

### Immunoblotting

The harvested cells were lysed in RIPA buffer containing protease and phosphatase inhibitors (Merck Millipore). The protein concentrations from the cell lysate, separate cytosol, and nuclear lysate were determined by a BCA Protein Assay Kit (Merck Millipore). Sixty micrograms of protein per well were subjected to SDS-PAGE. After electrophoresis, the proteins were transferred to a nitrocellulose membrane. The transferred membranes were blocked in 5% (w/v) nonfat dry milk or 5% (w/v) BSA in TBS (0.5 M NaCl, 20 mM Tris-HCl, pH 7.4) with 0.1% (v/v) Tween 20 and probed for the first antibody, followed by incubation with a secondary antibody conjugated with horseradish peroxidase (anti-rabbit, Cell Signaling; anti-mouse, Cell Signaling, Danvers, MA, USA) with visualization by ECL (Merck Millipore) with photographic film development. The first antibodies used in this study were anti-GAPDH (Cell Signaling, #5174), anti-Bim (Cell Signaling, #2933), anti-Bcl2 (Santa Cruz Biotechnology, sc-7382), anti-Bcl-xl (Cell Signaling, #2762), anti-caspase-3(a) (Cell Signaling, #9661), anti-PARP (Cell Signaling, #9542), anti-p21 (Cell Signaling, #2947), anti-Puma (Cell Signaling, #4976), and anti-p53 (Santa Cruz Biotechnology, sc-126). Immunoblot images were quantitated by Image Studio Lite (LI-COR Biosciences, Lincoln, NE, USA).

### Cell survival assay

CellTiter-Glo Luminescent (Promega, Madison, WI, USA) was used to evaluate cell death after compound treatment. About 1.5 × 10^4^ cells/per well were seeded on 96-well plates for 24 h. The old medium was replaced by a fresh medium with various concentrations of compounds singly or in combinations for 24 h. Then, the medium was sucked out and 50 μl/per well of CellTiter-Glo Luminescent solution was added to the 96-well plates. The plate contents were mixed for 2 min on a shaker and incubated at room temperature for 10 min. Luminescence was recorded by a luminometer.

### Cytosol and nuclear fractionation

Three plates of U2OS cells treated by DOX were harvested and then washed in hypotonic buffer (10 mM Hepes pH7.9, 1.5 mM MgCl_2_, 10 mM KCl, 0.5 mM DTT) with protease and phosphatase inhibitors. The washed cell pellets were resuspended in the hypotonic buffer for 10 min to swell cells. The swollen cells were homogenized by 5 up-and-down pushes through the syringe with 26 1/2 needles. The nuclei were spun down by centrifuging for 15 min at 4000 rpm. After spinning down, the cytosol supernatant and the collected nuclei were lysed in RIPA buffer (25 mM Tris-HCl pH7.6, 150 mM NaCl, 1% NP40 1 mM DTT, 0.1% NP-40, 1% sodium deoxycholate, 0.1% SDS) containing protease and phosphatase inhibitors. Both cytosol and the nuclei lysates were analyzed by immunoblotting.

### Statistical analysis

A paired *t* test using JMP13 determined the statistical significance of the results. *P < 0.05 or **P < 0.01 was considered significant.

## Data Availability

All data generated or analyzed during this study are included in this article
